# Metabolomics approach reveals annual metabolic variation in roots of *Cyathula officinalis* Kuan based on gas chromatography–mass spectrum

**DOI:** 10.1186/s13020-017-0133-1

**Published:** 2017-05-03

**Authors:** Kai Tong, Zhao-ling Li, Xu Sun, Shen Yan, Mei-jie Jiang, Meng-sheng Deng, Ji Chen, Jing-wei Li, Meng-liang Tian

**Affiliations:** 1 College of Agronomy, Sichuan Agricultural University, Chengdu, 611130 People’s Republic of China; 2 Maize Institute, Sichuan Agricultural University, Chengdu, 611130 People’s Republic of China; 3 Institute for New Rural Development, Sichuan Agricultural University, 608 Room, No. 1 building, 211 Huiming Road, Wenjiang District, Chengdu City, 611130 Sichuan Province People’s Republic of China

**Keywords:** Metabolomics, *Cyathula officinalis*, GC–MS, Harvest time, PCA, PLS-DA, Multivariate analysis, Herbal quality

## Abstract

**Background:**

Herbal quality is strongly influenced by harvest time. It is therefore one of crucial factors that should be well respected by herbal producers when optimizing cultivation techniques, so that to obtain herbal products of high quality. In this work, we paid attention on one of common used Chinese herbals, *Cyathula officinalis* Kuan. According to previous studies, its quality may be related with growth years because of the variation of several main bioactive components in different growth years. However, information about the whole chemical composition is still scarce, which may jointly determine the herbal quality.

**Methods:**

*Cyathula officinalis* samples were collected in 1–4 growth years after sowing. To obtain a global insight on chemical profile of herbs, we applied a metabolomics approach based on gas chromatography–mass spectrum. Analysis of variance, principal component analysis, partial least squares discriminant analysis and hierarchical cluster analysis were combined to explore the significant difference in different growth years.

**Results:**

166 metabolites were identified by using gas chromatography–mass spectrum method. 63 metabolites showed significant change in different growth years in terms of analysis of variance. Those metabolites then were grouped into 4 classes by hierarchical cluster analysis, characterizing the samples of different growth ages. Samples harvested in the earliest years (1–2) were obviously differ with the latest years (3–4) as reported by principal component analysis. Further, partial least squares discriminant analysis revealed the detail difference in each growth year. Gluconic acid, xylitol, glutaric acid, pipecolinic acid, ribonic acid, mannose, oxalic acid, digalacturonic acid, lactic acid, 2-deoxyerythritol, acetol, 3-hydroxybutyric acid, citramalic acid, *N*-carbamylglutamate, and cellobiose are the main 15 discrimination metabolites between different growth years.

**Conclusion:**

Harvest time should be well considered when producing *C. officinalis.* In order to boost the consistency of herbal quality, *C. officinalis* is recommended to harvest in 4th growth year. The method of GC–MS combined with multivariate analysis was a powerful tool to evaluate the herbal quality.

**Electronic supplementary material:**

The online version of this article (doi:10.1186/s13020-017-0133-1) contains supplementary material, which is available to authorized users.

## Background

Lots of Chinese herbs are recommended to harvest in fixed time period due to the variation of bioactive components during different cultivated years or different sampling seasons, such as *Alpinia oxyphylla* [1], *Hydrastis canadensis* [2], *Salvia miltiorrhizae* [3], *Sphallerocarpus gracilis* [4]. Uncontrolled metabolic variation has risk to decrease the herbal quality, which is contributed by the whole specific chemical profile [5, 6]. Hence, related study about chemical variation in different harvest time is valuable to establish the good agriculture practice (GAP) standards of Chinese traditional herbs in China [7].

As one of the most frequently-used traditional Chinese herbs [8], the roots of *Cyathula officinalis* Kuan have effects on anti inflammation [9], antioxidation [10], immune-enhancing [11], etc. and usually used to treat related diseases such as osteoarthritis [12], rheumatism [13] and chronic bacterial prostatitis [14] when combined with other herbs.

Historically, the cultivated *C. officinalis* is prior to harvest in the 3rd year after sowing by farmers in the main producing areas in China, like Sichuan province, China. However, without authoritative standards and powerful enforcement, herbal producers used to freely gather *C. officinalis* during 2–4 growth years in terms of herbal price fluctuation. Previous studies reported that several main bioactive compounds in *C. officinalis*, such as cyasterone, sengosterone, scoparone, daidzin and purerarin, varied significantly in different growth years [15]. Those results supported that fixed harvest year should be well considered on *C. officinalis* cultivation as one of important quality control factors, with an attempt to boost the consistency of different batches herbs or to obtain herbs with satisfied content of target components.

Recently, many literatures have illustrated that metabolomics approach is an efficient tool to evaluate herbal quality or discriminate easy-confused samples [16, 17]. This “omic” technique [18] provided us more comprehensive insight into the metabolic profile of herbs [19, 20]. Metabolomics approach based on LC–MS has been used to explore chemical difference of *C. officinalis* sampled from different areas [21]. However, besides of several main bioactive components, information about the total chemical composition of *C. officinalis* in different growth years is still scarce. In this study, we investigated the *C. officinalis* with different growth years by the GC–MS metabolomics platform. Analysis of variance (ANOVA), principal component analysis (PCA), partial least squares discriminant analysis (PLS-DA), hierarchical cluster analysis (HCA) data analysis methods were combined to measure annual metabolic variation in roots of *C. officinalis*, with the aim to finally facilitate the quality control of herbs.

## Methods

### Plant materials

The experimental plants were sowed among four successive years (2011–2014) in Baoxing country, Ya’an city, Sichuan province, China. In March, 2015, they were simultaneously collected. In total, 32 batches of authentic roots of *Cyathula officinalis* Kuan were identified by prof. Meng-liang Tian at College of Agronomy, Sichuan Agricultural University, where we deposited the voucher specimens of *C. officinalis*. All the plant materials were grew in a same farm by QiXiang farmer professional cooperative with same cultivated techniques before this study. We sorted these roots into 4 groups in terms of their growth years. They were labeled group A, group B, group C, and group D, which denoted that they had been grown for 1, 2, 3 and 4 years until sampling. Each group was composed of 8 biological replicates and each replicate was named by a unique sample identifier, which combined with group label and random number, like A1 or D8 (details see in Additional file 1). After washing the roots with pure water, all samples were immediately frozen in liquid N_2_ and stored at −80 °C until processing.

### Sample preparation

Samples for HPLC detection were prepared based on Ref. [8] with little modification. 1000 mg dried root powder was accurately weighted and then extract by methyl alcohol (20 ml) in ultrasonic for 30 min. Each sample group contained 8 replicates and each extraction repeated 3 times. Before HPLC analysis, extracting solutions were filtrated by 0.45 μm nylon membrane filter and diluted with equal amount of water.

Samples for GC–MS detection were prepared as follow: 100 mg fresh root tissue of each sample was accurately weighed and mixed with 0.4 ml methanol-chloroform (v/v; 3:1) and vortex for 10 s. 20 μl ribitol (0.2 mg/ml, stock in dH_2_O) was added in mixtures as internal standard. Mixtures were homogenized in ball mill (JXFSTPRP-24, Shanghai jinxin industrial development Co., Ltd) for 5 min at 55 Hz, subsequently centrifuged at 12,000 rpm for 15 min at 4 °C. The supernatant (approximately 0.4 ml) was transferred to a new GC/MS glass vial. The extracts were dried in a vacuum concentrator without heating for about 1.5 h. 80 μl methoxymethyl amine salt (dissolved in pyridine, final concentration of 20 mg/ml) was added into dried metabolites, afterwards incubated at 80 °C for 20 min in an oven after mixing and sealing. After that, 100 μl BSTFA (containing 1% TCMS, v/v) was added into each sample and incubated at 70 °C for an hour. When sample cooled to room temperature, 10 μl FAMEs (Standard mixture of fatty acid methyl esters, C8–C16:1 mg/ml; C18–C30:0.5 mg/ml in chloroform) was added to it, and finally mixed well for GC–MS detection.

### HPLC parameters

The cyasterone assaying used below parameters: Chromatographic column: Agilent Zorbax Eclipse XDB-C18 (5 μm particles, 4.6 mm × 150 mm). Flow rate: 0.8 ml/min. Temperature: 35 °C. Determine wavelength: 243 nm. Mobile phase: water and acetonitrile. Isocratic elution: 0–20 min, 18% water.

### GC–MS detection

Gas chromatography–mass spectrum analysis was performed using an Agilent 7890 gas chromatograph system coupled with a Pegasus HT time-of-flight mass spectrometer. The system utilized a DB-5M Scapillary column coated with 5% diphenyl cross-linked with 95% dimethylpolysiloxane (30 m × 250 μm inner diameter, 0.25 μm film thickness; J&W Scientific, Folsom, CA, USA). A 1 μl aliquot of the analyte was injected in splitless mode. Helium was used as the carrier gas, the front inlet purge flow was 3 ml/min, and the gas flow rate through the column was 20 ml/min. The initial temperature was kept at 50 °C for 1 min, then raised to 330 °C at a rate of 10 °C/min, then kept for 5 min at 330 °C. The injection, transfer line, and ion source temperatures were 280, 280, and 250 °C, respectively. The energy was −70 eV in electron impact mode. The mass spectrometry data were acquired in full-scan mode with the m/z range of 30–600 at a rate of 20 spectra per second after a solvent delay of 366 s. Before statistical analysis, the GC–MS method was validated by internal standard compound (Ribitol), whose standard deviation of retention time was 0.014 (n = 32).

### Statistical analysis

Chroma TOF 4.3X software of LECO Corporation and LECO-Fiehn Rtx5 database were used for raw peaks exacting, the data baselines filtering and calibration of the baseline, peak alignment, deconvolution analysis, peak identification and integration of the peak area [22]. The RI (retention time index) method was used in the peak identification, and the RI tolerance was 5000. Metabolite data were normalized by dividing each peak area value by the area of internal standard (Ribitol). After that, the data were log10 transformed, mean-centered and divided by the standard deviation of each variable before performing statistical analysis. All the statistical analyses, such as ANOVA, PCA, PLS-DA, HCA, were performed by using MetaboAnalyst 3.0 [23]. The Minimum Standards of Reporting Checklist contains details of the experimental design, and statistics, and resources used in this study (Additional file 2).

## Results

### HPLC detection

As the only certificated quality marker by Chinese Pharmacopoeia (edition 2015) [8], cyasterone was detected in four groups by HPLC. The results showed that the content of cyasterone in each sample could meet the minimum requirement (≥0.030%) of Chinese Pharmacopoeia. However, the 4-years growth ages plants have the highest content of cyasterone (0.087%), followed by 3-years group (0.076%), 2-year group (0.065%) and 1-year group (0.039%) (details see Additional file 3). These results were also partial proved by previous studies [15]. Therefore, considered the content of marker compound, herbs with 1–4 growth years ages were all qualified and the 4th year was the best harvest year.

### GC–MS data extraction

**Fig. 1:**
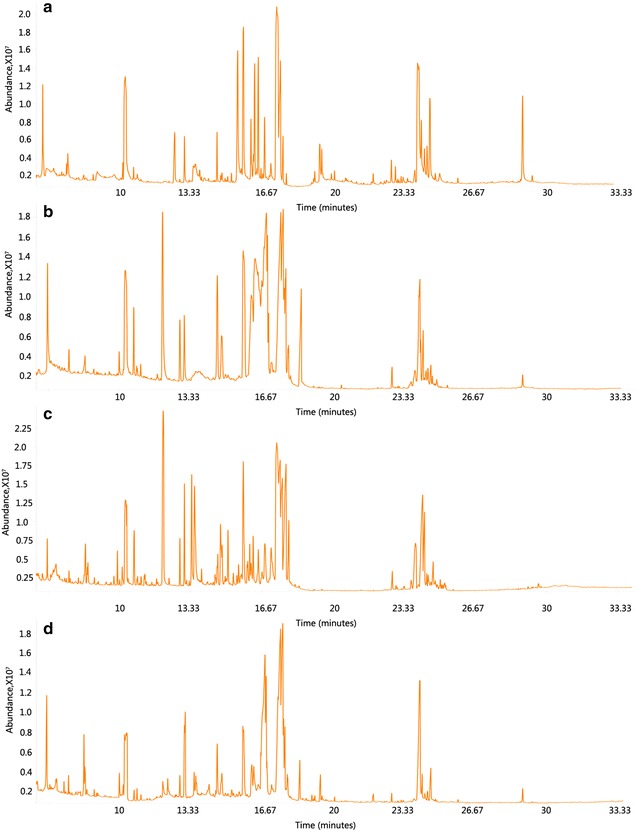
Representative GC–MS chromatogram of each group. **a** Sample with 1 growth age. **b** Sample with 2 growth ages. **c** Sample with 3 growth ages. **d** Sample with 4 growth ages

To obtain an overview of annual metabolic changes in roots of *C. officinalis*, we carried on the GC–MS approach to all samples. The representative GC–MS chromatograms showed obvious variation in different growth ages (Fig. 1a–d). In total, 752 chromatographic peaks were detected and then numbered 1–752 in sequence of retention time. Peak 442 was contributed by ribitol as reference substance. In order to remove the systematic noise, we just extracted those peaks that were successfully discovered (peak area value >0) at least 6 times in either groups (n = 8). In result, 341 peaks (not include peak 442) were retained. Among them, 166 peaks were given identified chemical name (details see Additional files 1, 4). It should be noted that 4 chemicals were identified twice. They were 3-hydroxypropionic acid (peak 99 and 107), aspartic acid (peak 295 and 349), xylitol (peak 431 and 432), and diglycerol (peak 446 and 457). This may result from the insufficient precision of methods. To the end, the final dataset was composed of all the 166 peaks of 32 samples and their peak area values, which was used to the following ANOVA, PCA, HCA, PLS-DA etc.

### One-way ANOVA

**Fig. 2:**
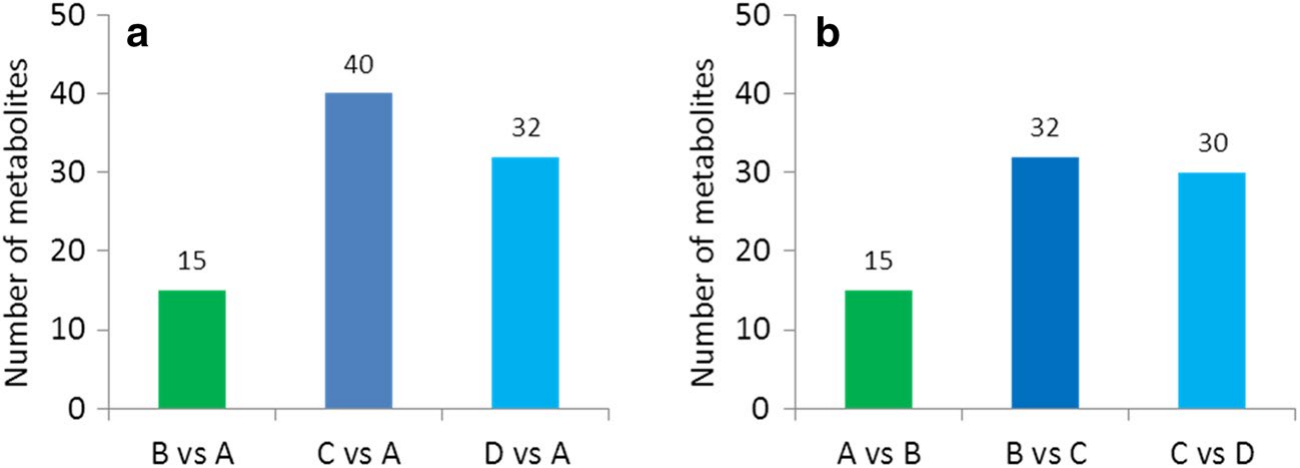
Number of metabolites that changed significantly. **a** *Each column* shows the number of significantly changed metabolites (*P* < 0.05, ANOVA) with respect to the first growth year for the next 3 groups. **b** *Each column* shows the number of significantly changed metabolites (*P* < 0.05, ANOVA) with respect to the previous growth year for group B, group C and group D

We firstly carried on univariate analysis method, ANOVA, to have an overview of all 166 metabolites data, attempted to simply find potentially important metabolites. In result, 63 metabolites showed potentially significant difference about their content between 4 groups using the standard *P* < 0.05 (detail see Additional file 5). Compared with the 1st growth year (group A), 15 metabolites changed significantly when herbs grew for two years (group B), while 40 metabolites changed in 3rd year (group C) and 32 metabolites in 4th year (group D). This trend was as well illustrated in Fig. 2a, which clearly showed us most metabolic variations happened in 3rd year after sawing. Similar trend was also revealed in Fig. 2b by comparing the number of metabolites changed significantly between previous year and following year. Therefore, we speculated that the 3rd year, when the most large-scale of metabolites changed, more likely a turning point for *C. officinalis* metabolism.

**Fig. 3:**
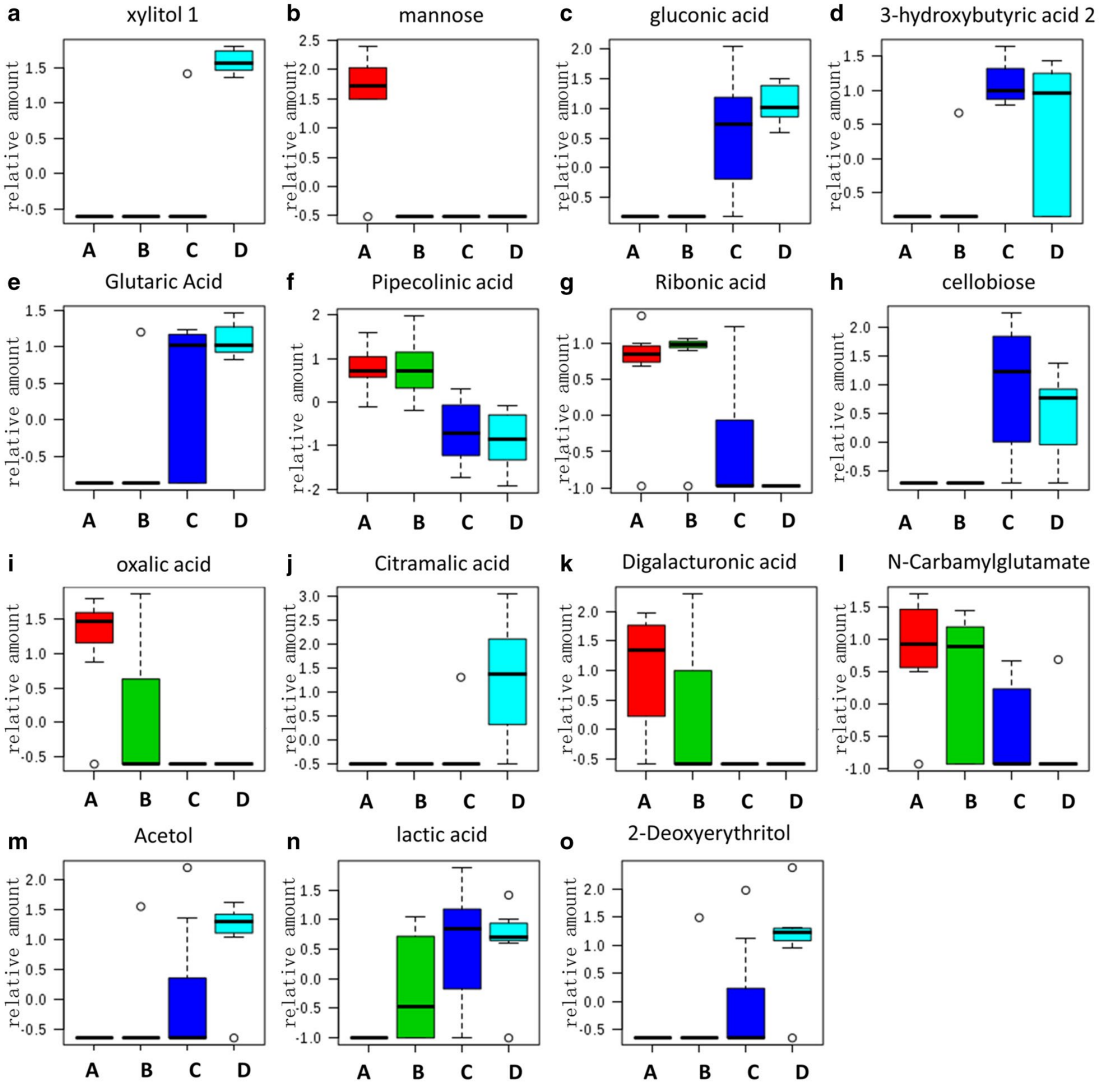
Typical metabolite variation in different growth years. **a**–**j** Top 10 potential biomarkers determined by ANOVA (*P* < 0.000 1). **a**–**o** Top 15 potential biomarkers determined by VIP scores

Top 10 typical metabolites were listed Additional file 5, using the standard of *P* < 0.0001. In Fig. 3 their changes in different growth years were illustrated by box plots, which showed that those metabolites more or less prone to accumulate in some certain years. For instance, xylitol and citramalic acid were hardly detected in the first 3 growth years until grew for 4 years. While mannose and oxalic acid accumulated rich in fist year but decreased during the 2–4 years. Gluconic acid, 3-hydroxybutyric acid, glutaric acid and cellobiose were easily detected in 3–4 years while little existed in 1–2 years. However, this trend was inverted for pipecolinic acid and ribonic acid.

### Cluster analysis

**Fig. 4:**
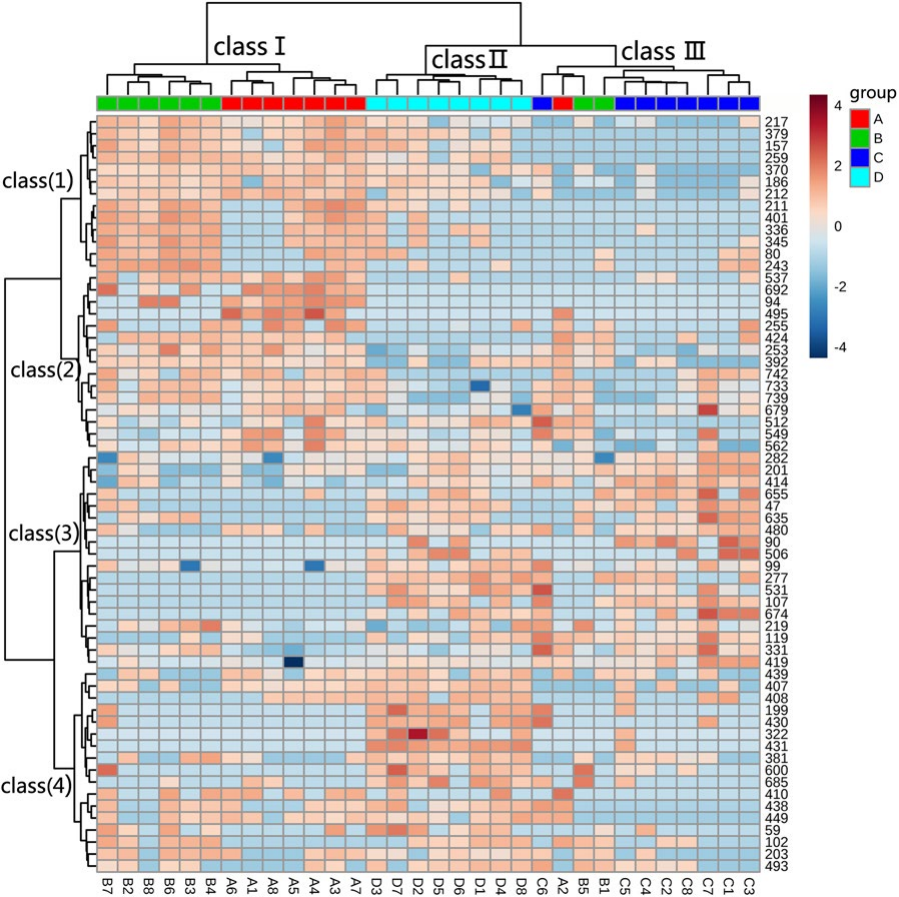
Significantly changed metabolites in each sample based on ANOVA

To have a further visually insight on every metabolite content change in different growth years. We performed a heat-map combined with HCA, using extracted dataset composed by 63 metabolites filtered by ANOVA (*P* < 0.05). As illustrated in Fig. 4, 32 samples trended to separate into 3 classes. Samples with same growth age had trend to cluster in same class. For instance, samples of group C all clustered in class III and group D all clustered in class II. Seven samples from group A and 6 samples from group B were together clumped in class I and further separated into two subclasses. Unusually, the rest 3 samples of group A or B, A2, B1, B5, were together put into class III. This means that those 3 samples were more similar to group C. The fact that only group D was clustered in an independent class, class II, indicated that its metabolic profile was stabilized when herbs of 4 growth age. Therefore, compared with the traditional cultivation, more proper harvest time for *C. officinalis* may be the 4th year after sowing because of the micro-change in metabolic profile between samples.

Showed in Fig. 4, 4 metabolite classes were found in the heat-map, each of which revealed the content distribution in different sample classes or growth years. Metabolites in Class (1), like glycine (peak 217), 2-hydroxy-3-isopropylbutanedioic acid (peak 379), valine (peak 157), were least abundant in 3 growth years than any other growth ages. However, metabolites in class (4), like 2-deoxyerythritol (peak 199), acetol (peak 430), citramalic acid (peak 322), were most abundant in 4 growth years. Most of metabolites in Class (2), like ribonic acid (peak 424), pipecolinic acid (peak 253), *N*-carbamylglutamate (peak 537), had high content in the 1–2 growth years compared with the next 3 or 4 growth years, while metabolites in class (3), like gluconic acid (peak 618), 3-hydroxypropionic (peak 99), glutaric acid (peak 277), cellobiose (peak 674), lactic acid (peak 47), trended to accumulate in 3–4 growth years when compared with 1–2 growth years.

### PCA

**Fig. 5:**
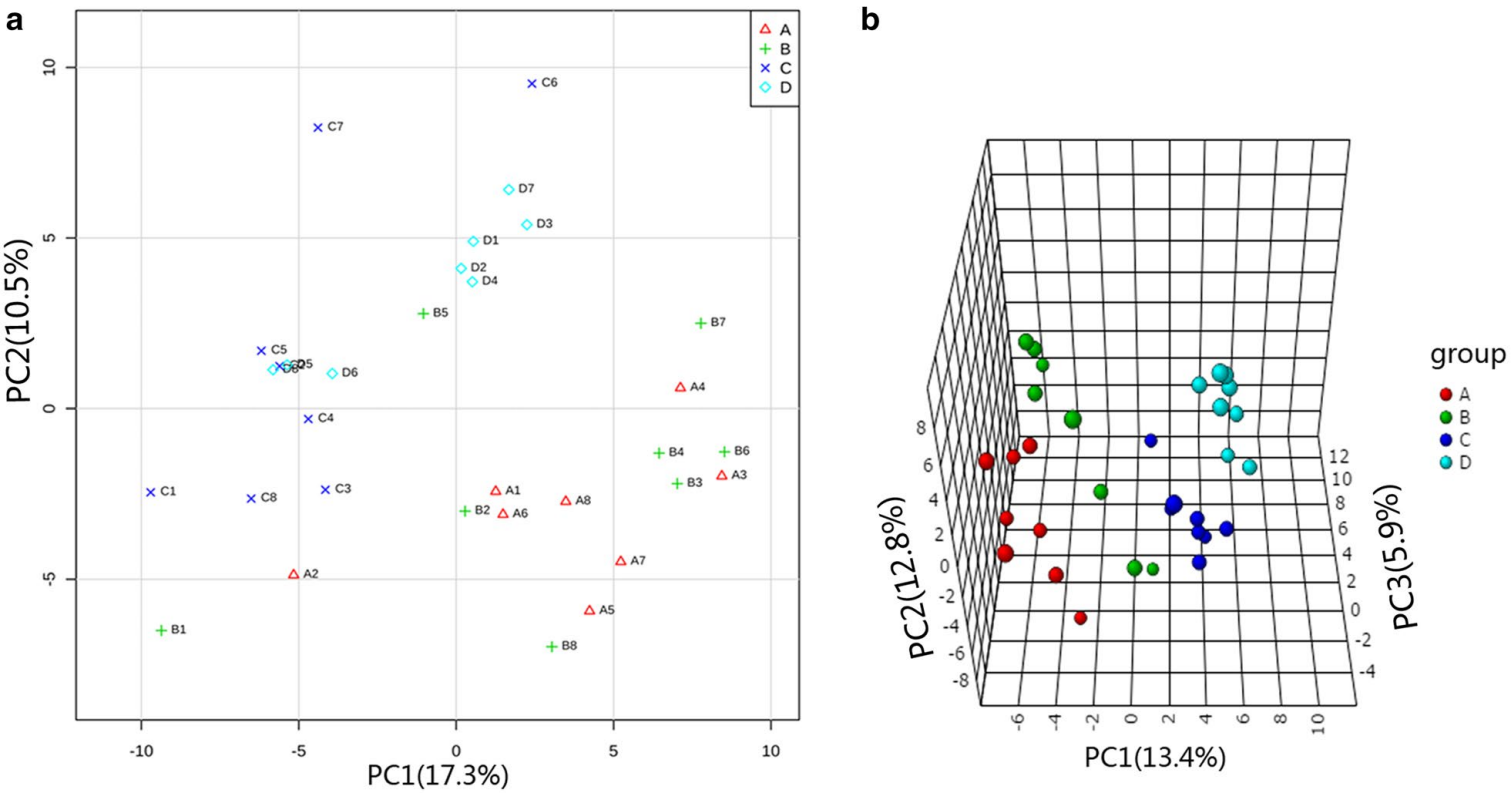
Score plot of PCA and PLS-DA. **a** PCA score plot based on PC1 and PC2. **b** PLS-DA score plot with respect to the first three PCs

As one of common used unsupervised methods of multivariate analysis, we performed PCA on the 166 metabolites dataset. Considering only the first two principal components, PC1 and PC2, explained 27.8% variance. An obvious separation between the earliest (group A and group B) and latest (group C and group D) growth years in Fig. 5a was discovered, which exhibited the notable metabolic difference between them. The considerable overlap between the group A and group B indicated their metabolic profiles were similar. This fact as well had been told by Fig. 4, where group A and group B were clustered in two subclasses although belonged to a same upper class. Compared with earliest growth years, the latest groups, group C or group D, were better separated due to samples of group D gathered more closely in this PCA score plot. This result proved again that group D had less internal difference relatively.

### PLS-DA

**Fig. 6:**
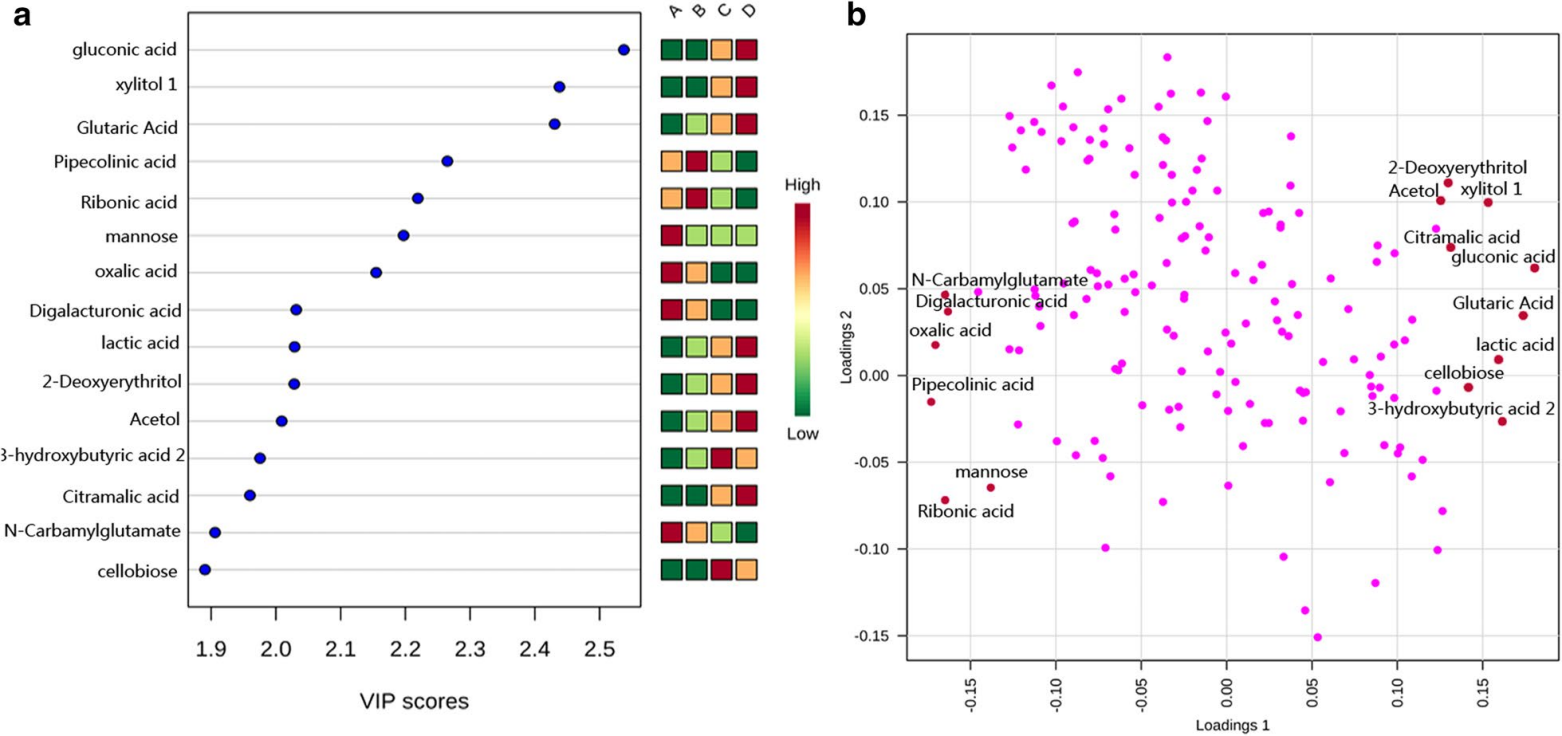
Potential chemical markers. **a** Top 15 metabolites selected by VIP score. **b** Loading plot of PLS-DA regression model

**Fig. 7:**
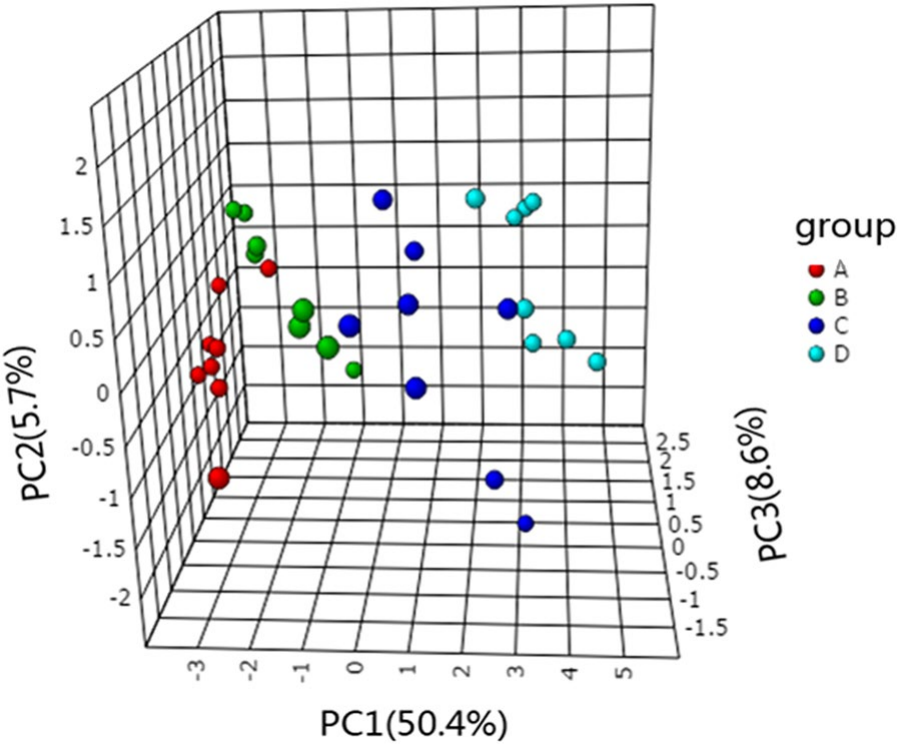
PLS-DA score plot built by top 15 metabolites dataset

Principal component analysis analysis demonstrated the presence of discriminating factors which allowed the separation between earliest growth years and latest years. However, this kind of unsupervised method did not allow observing well separation of every two groups, such as group A/group B or group C/group D. To verify whether our current metabolites dataset provided enough information could detail the significant difference of any two groups, we decided to use a supervised method as PLS-DA. In result, this method dose exhibited the ability to discriminate each group in three dimensional score plot with three principle components (Fig. 5b), accounting for 32.1% variance. As showed in Fig. 5a, b, the main factor that drived the separation between each group was PC1. In order to summarize the importance of metabolites for constructing PC1, we listed the top 15 metabolites with high variable influence on projection (VIP) score (Fig. 6a). They were gluconic acid, xylitol, glutaric acid, pipecolinic acid, ribonic acid, mannose, oxalic acid, digalacturonic acid, lactic acid, 2-deoxyerythritol, acetol, 3-hydroxybutyric acid, citramalic acid, *N*-carbamylglutamate, and cellobiose. These results were correlated with main loadings of PLS regression (Fig. 6b). In order to verify whether those 15 metabolites have ability to discriminate different growth years as potential chemical markers, we re-performed PLS-DA based on 15 metabolites. Luckily, the new score plot (Fig. 7) who produced by this simple dataset was very similar with the score plot produced by 166 metabolites (Fig. 5b). That means, using the 15 metabolites to evaluate the quality of herbs is feasible.

## Discussion

Unlike LC–MS or NMR, GC–MS we used in the present study prefers to offer information about primary metabolites, such as sugar, protein, lipid and organic acids et al. Generally, primary metabolites could be the precursors for the secondary metabolites, like various terpenoids including cyasterone. In the present study, a wild range of variation of primary metabolites that related sugar metabolism showed regular change in different growth years, like mannose, acetol and d-glyceric acid (detail see Additional file 3). Those metabolites related sugar metabolism pathway could further offer some important metabolic intermediates, like pyruvic acid and phosphoglyceraldehyde, which were used in the biosynthesis pathways of terpenoids [24].

In the current Chinese Pharmacopoeia, one of the most important methods for herb quality control is to evaluate the content of one or more chemical compounds. However, this method ignores the synergistic effect of multiple compounds, which is much emphasized in clinical application of traditional Chinese medicine [25]. Therefore, a better method to evaluate the herbal quality is to characterize the metabolic profile with a wide range of chemical compositions, other than just simply assessing one or several chemical compounds. Generally, herbs with a similar metabolic characterization would have similar properties. In the present study, 166 metabolites were synergistically used to investigate the herbal quality and successfully revealed the unique chemical patterns of samples with different growth ages. Compared with the single chemical marker assessing, this method provided more chemical information of plants in an overall perspective.

Based on current analytical techniques, it is impossible to obtain the truly whole compositions of medicinal plants. Ideally, the represented metabolites are unique components that contribute to the therapeutic effects of herbal medicines. However, for many plants, the accurate pharmacological compounds are still unclear. Therefore, other chemical components that is of interest for quality control purposes are also used to as markers. As the different role on quality control, those markers could further be classified into eight categories regardless whether they are active compounds [26]. In the present study, we selected 15 metabolites as potential chemical markers for quality control mainly because of their ability to evaluate the herbal consistency and discriminate the different growth ages.

Quality control of herbal medicines aims to ensure their authentication, consistency, safety and efficacy. As the variation on quality control purpose, various chemical markers or analysis techniques should be used at different conditions. In this study, we established a GC–MS approach to reveal the variation on *C. officinalis* with different growth ages and found out several marker compounds to discriminate them. However, it should be noted that herbal quality is affected by climate factors, like precipitation, sunlight, temperature et al., which were not actually measured in this study. Therefore, in order to test the discrimination efficacy of these markers, followed multi-plot demonstration for several years is recommended.

## Conclusions

The results mentioned above showed that the chemical profile of *C. officinalis* could be quite different when collected in different growth years. Gluconic acid, xylitol, glutaric acid, pipecolinic acid, ribonic acid, mannose, oxalic acid, digalacturonic acid, lactic acid, 2-deoxyerythritol, acetol, 3-hydroxybutyric acid, citramalic acid, *N*-carbamylglutamate, and cellobiose are the main 15 discrimination metabolites between different growth years. With the aim to boost the consistency of herbal quality, *C. officinalis* is recommended to harvest in 4th growth year based on the information provided by GC–MS. The method of GC–MS combined with multivariate analysis is a powerful tool to discriminate the different herbal growth age.

## Additional files

**Additional file 1.** The dataset composed by all metabolite peak area values of 32 samples.

**Additional file 2.** Minimum Standards of Reporting Checklist.

**Additional file 3.** The amount of cyasterone in different growth years.

**Additional file 4.** Metabolites identified by GC–MS.

**Additional file 5.** Metabolites changing significantly between groups based on ANOVA results.

## Abbreviations

GC–MS: gas chromatography–mass spectrum
GAP: good agriculture practice
LC–MS: liquid chromatography–mass spectrum
HPLC: high performance liquid chromatography
NMR: nuclear magnetic resonance
ANOVA: analysis of variance
PCA: principal component analysis
PLS-DA: partial least squares discriminant analysis
HCA: hierarchical cluster analysis
PC: principal component
VIP: variable influence on projection
