# Supplementary material for: Metabolomics approach reveals annual metabolic variation in roots of Cyathula officinalis Kuan based on gas chromatography–mass spectrum
Source: Chin Med. 2017 May 3;12:12. doi: 10.1186/s13020-017-0133-1 (PMC5414129; doi:10.1186/s13020-017-0133-1)

Additional file 3. The amount of cyasterone in different growth years.

	1 year	*2 years*	*3 years*	*4 years*
Sample 1	0.034%	0.061%	0.069%	0.088%
Sample 2	0.030%	0.068%	0.088%	0.082%
Sample 3	0.041%	0.071%	0.081%	0.091%
Sample 4	0.036%	0.059%	0.072%	0.107%
Sample 5	0.042%	0.053%	0.081%	0.078%
Sample 6	0.032%	0.074%	0.065%	0.082%
Sample 7	0.044%	0.067%	0.074%	0.079%
Sample 8	0.051%	0.065%	0.078%	0.091%
**Average**	**0.039%**	**0.065%**	**0.076%**	**0.087%**

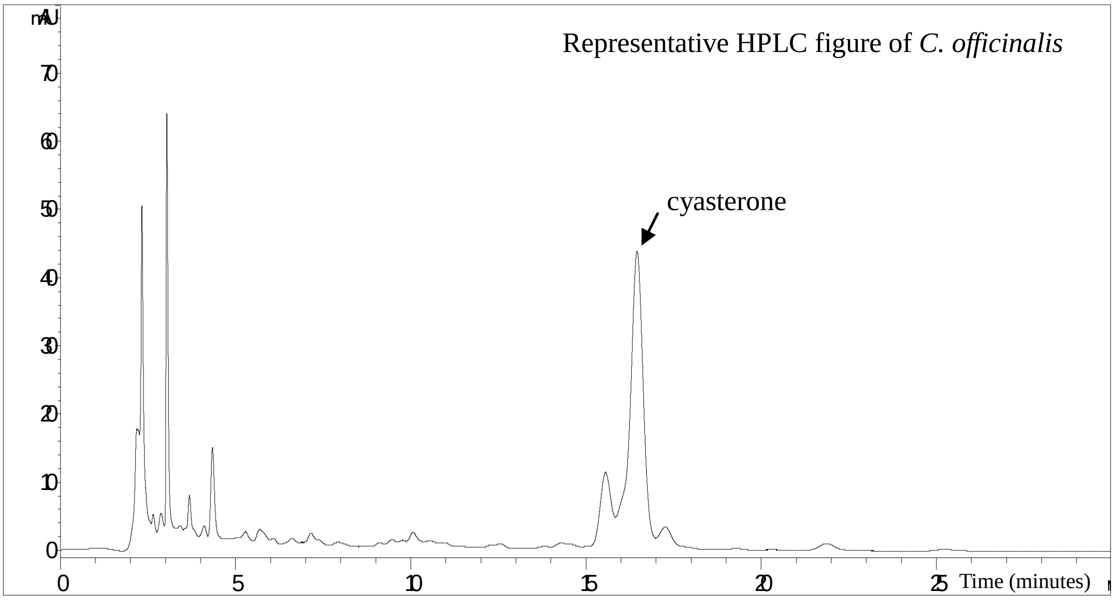

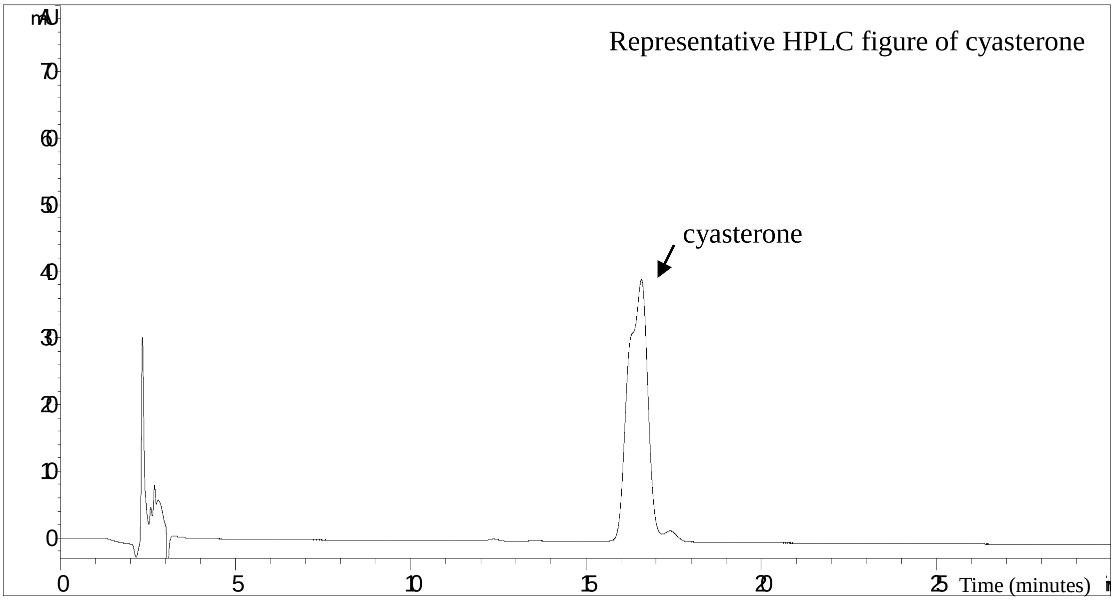

Supplement: Supplementary file 3 — Additional file 3. The amount of cyasterone in different growth years. [file 13020_2017_133_MOESM3_ESM.pdf]
